# Large-area flat-panel detector versus conventional detector for full-spine digital radiography: a retrospective comparative study

**DOI:** 10.3389/fmed.2026.1883819

**Published:** 2026-09-01

**Authors:** Fuqiang Lin, Yuting Gao, Wei Deng, Hong Ren

**Affiliations:** 1 Department of Radiology, Sir Run Run Shaw Hospital, Zhejiang University School of Medicine, Hangzhou, China; 2 Department of Radiology, Hospital of Zhejiang People's Armed Police, Hangzhou, China

**Keywords:** digital radiography, flat-panel detector, full-spine imaging, image quality, spinal radiography

## Abstract

**Objective:**

To compare workflow efficiency, tube current-exposure time product (mAs), and image quality between a DRX-LC large-area flat-panel detector and a conventional flat-panel detector for full-spine digital radiography.

**Methods:**

In this retrospective comparative study, radiographs and examination records of 90 adult patients who underwent full-spine anteroposterior (AP) and lateral (LAT) digital radiography between August and September 2025 were retrospectively reviewed. Patients were categorized according to the detector used during clinical imaging into a DRX group (Carestream DRX-LC, 42.58 × 120.87 cm) or a conventional group (33.0 × 43.2 cm), with 45 patients in each group. The DRX group underwent single-exposure full-spine imaging, whereas the conventional group underwent 4–6 segmented acquisitions followed by automatic stitching. Examination duration and mAs values for AP and LAT imaging were recorded. Image quality was independently assessed by four experienced reviewers using a 4-point scoring system, and mean scores were analyzed.

**Results:**

Baseline characteristics were comparable between the two groups (all *P* > 0.05). The DRX group had a significantly shorter examination duration than the conventional group (3.63 ± 0.83 min vs. 11.59 ± 2.31 min, *P* < 0.001). Subjective image quality scores were significantly higher in the DRX group [median [IQR], 4 [3–4] vs. 3 [2.5–3]; *P* < 0.001]. Compared with the conventional group, the DRX group also showed lower AP mAs (39.12 ± 1.16 vs. 70.43 ± 10.45, *P* < 0.001), lower LAT mAs (54.56 ± 3.02 vs. 76.69 ± 12.83, *P* < 0.001), and lower cumulative mAs (93.88 ± 3.59 vs. 146.89 ± 21.82, *P* < 0.001). The single-exposure DRX-LC images showed clearer visualization of vertebral cortices and trabeculae and fewer artifacts.

**Conclusion:**

For full-spine digital radiography, use of the DRX-LC large-area detector was associated with fewer exposures, shorter examination time, lower mAs requirements, and better subjective image quality. These findings support its clinical value for optimizing full-spine imaging workflows.

## Introduction

Full-spine anteroposterior (AP) and lateral (LAT) digital radiography is a core imaging method for evaluating spinal deformity, sagittal and coronal alignment, and degenerative spinal abnormalities because it enables visualization of the entire spine in the weight-bearing position and provides clinically actionable morphologic information (1, 2). As the core component of a digital radiography system, the effective imaging area of the flat-panel detector (FPD) directly influences single-exposure coverage and acquisition strategy (3, 4).

Conventional FPDs usually require multistation acquisition and image stitching to generate full-spine imaging. This approach increases workflow complexity, prolongs examination time, and may introduce stitching errors or motion-related artifacts. Repeated segmented acquisition also increases cumulative exposure output during a single examination (5, 6). In a pediatric scoliosis cohort, Supakul et al. reported stitching errors with potential for false diagnosis in 16% of digitally stitched examinations (7). In a Swiss comparison of standard digital radiography and biplanar slot-scanning imaging, whole-spine examination time was shorter with the biplanar system than with standard digital radiography (8). Studies have also shown that full-spine radiography protocols can be optimized while maintaining clinical image acceptability. In a Korean study of 223 patients, a 50% reduction in mAs did not significantly affect subjective image-quality scores or the ability to perform Cobb angle measurements in full-spine radiographs (9). However, previous studies have primarily evaluated dose-optimization strategies within stitched radiography or compared standard digital radiography with fundamentally different long-length imaging technologies. Their findings cannot be directly extrapolated to a comparison between a single-exposure large-area detector–based imaging system and a conventional segmented acquisition and stitching workflow.

In addition, clinical image quality is determined by the entire imaging pathway, including acquisition geometry, exposure-control strategy, scatter control, detector characteristics, and image processing, rather than detector size alone (10, 11). Comparative clinical evidence evaluating the Carestream DRX-LC system (Carestream Medical Equipment Co., Ltd., Shanghai, China) and a conventional FPD workflow in routine full-spine radiography remains limited. Therefore, this retrospective study compared examination duration, tube current-exposure time product (mAs), and subjective image quality between the DRX-LC large-area FPD–based imaging system and a conventional FPD imaging system.

## Methods and materials

### Study design and patients

This retrospective comparative study was approved by Sir Run Run Shaw Hospital, Zhejiang University School of Medicine, which waived the requirement for informed consent because of the retrospective design. Patients who underwent clinically indicated AP and LAT full-spine radiography at our institution between August and September 2025 were consecutively identified from the institutional imaging records. Of 102 potentially eligible patients, 12 were excluded because of nonstandard or incomplete AP/LAT examinations (*n* = 4), missing images or examination records (*n* = 3), repeated examinations during the study period (*n* = 2), or missing baseline data required for matching (*n* = 3). The remaining 90 patients were matched 1:1 between the DRX-LC and conventional imaging groups according to age, sex, and body mass index, resulting in 45 patients in each group.

### Imaging protocol

All examinations were performed on a DRX-CompassA digital radiography system equipped with a dedicated spine antiscatter grid. In the DRX-LC group, the source-to-image distance (SID) was 240 cm for both AP and LAT imaging. Tube voltage was set at 80 kV for AP imaging and 85 kV for LAT imaging, with tube current settings of 630 mA and 560 mA, respectively. In the conventional FPD group, the SID was 180 cm for both AP and LAT imaging. Tube voltage was set at 80 kV for AP imaging and 90 kV for LAT imaging, with tube current settings of 630 mA and 560 mA, respectively. A focal spot size of 1.2 mm was used in both groups. Automatic exposure control was used in both groups. Exposure duration was automatically determined according to patient attenuation and the preset detector exposure level; therefore, the recorded tube current-exposure time product values varied among patients and are reported in Table 1. For DRX-LC imaging, collimation was adjusted to encompass the full spine with minimal margins. For conventional imaging, each acquisition was collimated to the target spinal region, with an approximately 2.5-cm overlap between adjacent fields to permit image stitching.

**Table 1:** Comparison of baseline characteristics, examination duration, subjective image quality, and exposure parameters between the DRX-LC and conventional flat-panel detector groups.

Characteristic	DRX-LC large-area FPD group (*n* = 45)	Conventional FPD group (*n* = 45)	*P* value
Baseline characteristics			
Age, y	25.36 ± 9.98	21.64 ± 9.31	0.07
Sex, male/female, no.	31/14	25/20	0.28
Body mass index, kg/m^2^	20.58 ± 2.83	20.97 ± 2.12	0.45
Outcome measures			
Examination duration, min	3.63 ± 0.83	11.59 ± 2.31	<0.001
Subjective image quality, points	4 (3–4)	3 (2.5–3)	<0.001
Anteroposterior tube current- exposure time product, mAs	39.12 ± 1.16	70.43 ± 10.45	<0.001
Lateral tube current- exposure time product, mAs	54.56 ± 3.02	76.69 ± 12.83	<0.001
Cumulative tube current- exposure time product, mAs	93.88 ± 3.59	146.89 ± 21.82	<0.001

Data are means ± standard deviations unless otherwise indicated. Subjective image quality scores are presented as median (interquartile range) and were compared using the Mann–Whitney U test. Other continuous variables were compared using the independent-samples t test, and sex distribution was compared using the chi-square test. FPD, flat-panel detector; mAs, tube current-exposure time product.

The x-ray tube anode-cathode axis was aligned with the transverse short-axis dimension of the detector; therefore, the anode heel effect was oriented transversely rather than along the cranio-caudal full-spine imaging axis.

The DRX group underwent single-exposure imaging using the DRX-LC detector (42.58  ×  120.87 cm), which directly covered the entire spine. The conventional group was imaged with a standard detector (33.0 × 43.2 cm) using 4–6 segmented acquisitions with an approximately 2.5-cm overlap between adjacent fields, followed by automatic stitching at the workstation.

For AP imaging, patients stood upright with the back against the detector. For LAT imaging, patients were positioned in the left LAT orientation with the body closely apposed to the detector, and fixation straps were used when needed to reduce motion. The central ray was horizontally centered at the T12-L1 level in both projections. All images underwent a unified post-processing workflow and were transferred to the picture archiving and communication system. Examination duration was defined as the time from the initiation of patient positioning to completion of image post-processing. The time required for picture archiving and communication system transfer was not included.

### Image quality assessment

Image quality was assessed independently by four experienced reviewers, including one senior radiologic technologist, one associate chief radiologist, and two spine subspecialty associate chief radiologists. A 4-point scoring system was used, and the mean score across the four reviewers was entered into the statistical analysis (9, 12, 13).

**Figure 1:**
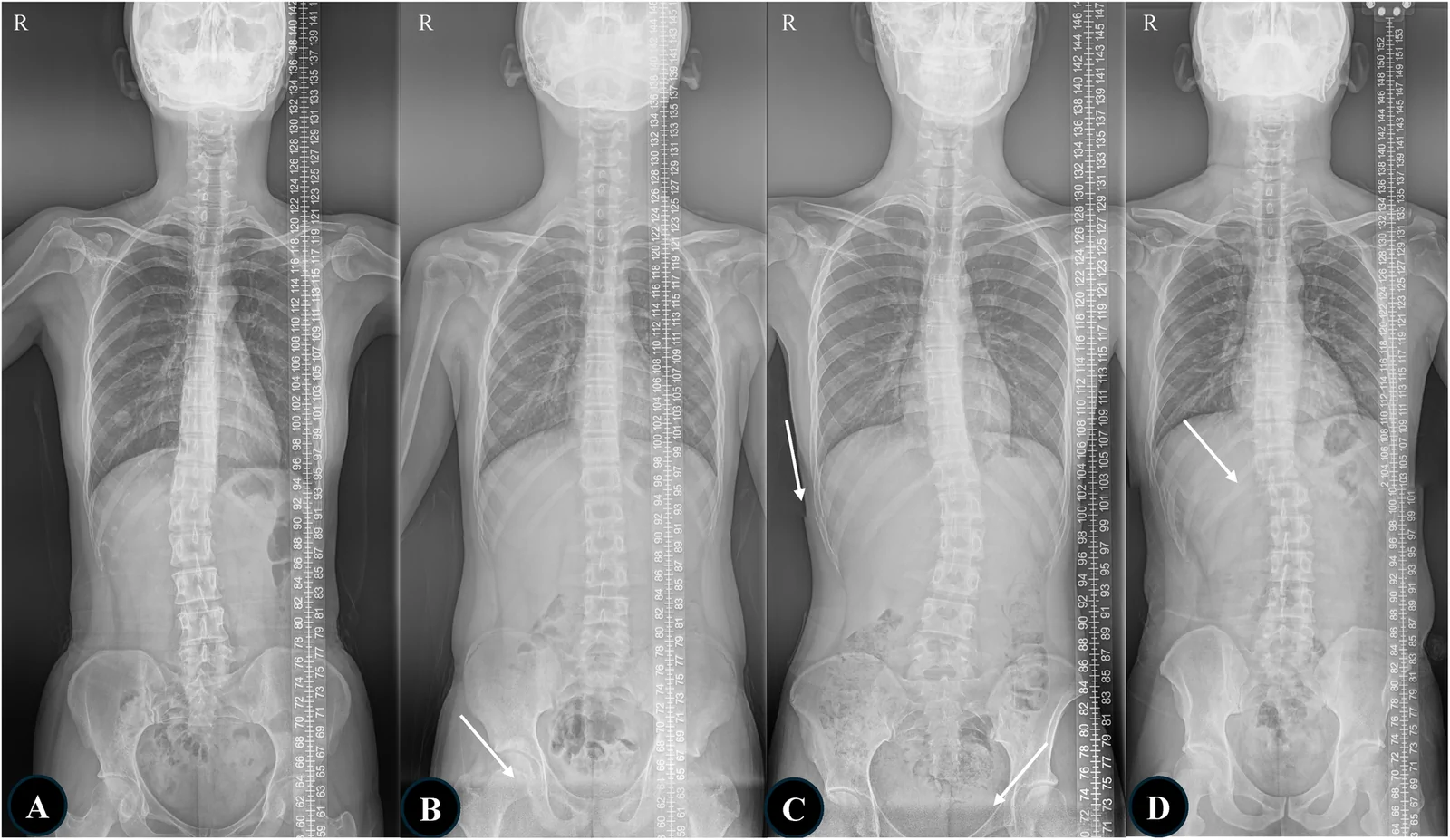
Representative full-spine anteroposterior radiographs illustrating the 4-point subjective image quality scoring system. **(A)** Score 4: all predefined image-quality criteria were satisfied. **(B)** Score 3: a stitching artifact was present (arrow) but did not affect diagnostic interpretation. **(C)** Score 2: stitching artifacts were present (arrows), including transverse misregistration at the rib level; however, the image remained diagnostically acceptable. **(D)** Score 1: severe stitching misregistration at the lumbar spine (arrow) substantially impaired diagnostic interpretation.

Image quality was evaluated using four predefined criteria: (1) patient positioning and full-spine coverage; (2) image density, contrast, and visualization of vertebral cortical and trabecular structures; (3) artifacts, including motion, stitching, and extraneous artifacts; and (4) appropriateness and noninterference of image markers. Each radiograph was assigned an overall 4-point image quality score on the basis of these criteria (Table 2). Representative images were shown in Figure 1. A higher score indicated better image quality. The mean score of the four reviewers was used for statistical analysis.

**Table 2:** Subjective image quality assessment criteria and overall 4-point scoring scale.

Item	Definition
Assessment criteria	**1. Positioning and coverage**: Correct patient positioning and adequate visualization of the entire spine. **2. Image quality and anatomic detail**: Appropriate image density and contrast, with clear visualization of vertebral cortices and trabecular structures. **3. Artifacts**: Absence of motion, stitching, extraneous, or contamination artifacts that could affect image interpretation. **4. Image markers**: Correct placement of image markers without obscuring relevant anatomy.
Score 4: Excellent	All four criteria were satisfied. The image was of excellent quality and fully suitable for diagnostic interpretation.
Score 3: Good	One criterion was suboptimal, but image quality remained adequate and diagnostic interpretation was unaffected.
Score 2: Acceptable	Two criteria were suboptimal, resulting in minor image-quality limitations but no substantial impairment of diagnostic interpretation.
Score 1: Poor	Three or more criteria were suboptimal, or any single limitation substantially impaired diagnostic interpretation.

### Statistical analysis

Statistical analysis was performed with SPSS version 22.0. Subjective image quality scores were treated as ordinal data. For patient-level group comparisons, the median score across the four reviewers was calculated for each radiograph and is presented as median (interquartile range). Groups were compared using the Mann–Whitney U test. Inter-rater agreement among the four reviewers was assessed using Kendall's coefficient of concordance (W). Other continuous variables are presented as mean ± standard deviation and were compared using the independent-samples t-test. Categorical variables were compared using the chi-square test. A two-sided *P* value of less than 0.05 was considered statistically significant. Because this was a retrospective study, no *a priori* sample size calculation was performed; all consecutive eligible patients during the predefined study period were included. To assess the adequacy of the available sample, a sensitivity analysis was performed for the primary outcome of subjective image quality score. With 45 patients per group, a two-sided alpha level of 0.05, and the sample provided 80% power to detect a standardized between-group difference of approximately 0.60. The observed between-group difference in image quality score corresponded to a standardized effect size of 1.22.

## Results

### Baseline characteristics

A total of 90 patients were included, with 45 in the DRX group and 45 in the conventional group. Baseline characteristics were comparable between the two groups (Table 1). The mean age was 25.36 ± 9.98 years in the DRX group and 21.64 ± 9.31 years in the conventional group (*P* = 0.07). The sex distribution did not differ significantly between groups (*P* = 0.28), with 31 men (68.89%) and 14 women (31.11%) in the DRX group and 25 men (55.56%) and 20 women (44.44%) in the conventional group. Mean body mass index was also similar between groups (20.58 ± 2.83 kg/m2 vs. 20.97 ± 2.12 kg/m^2^, *P* = 0.45).

### Examination duration and subjective image quality

**Figure 2:**
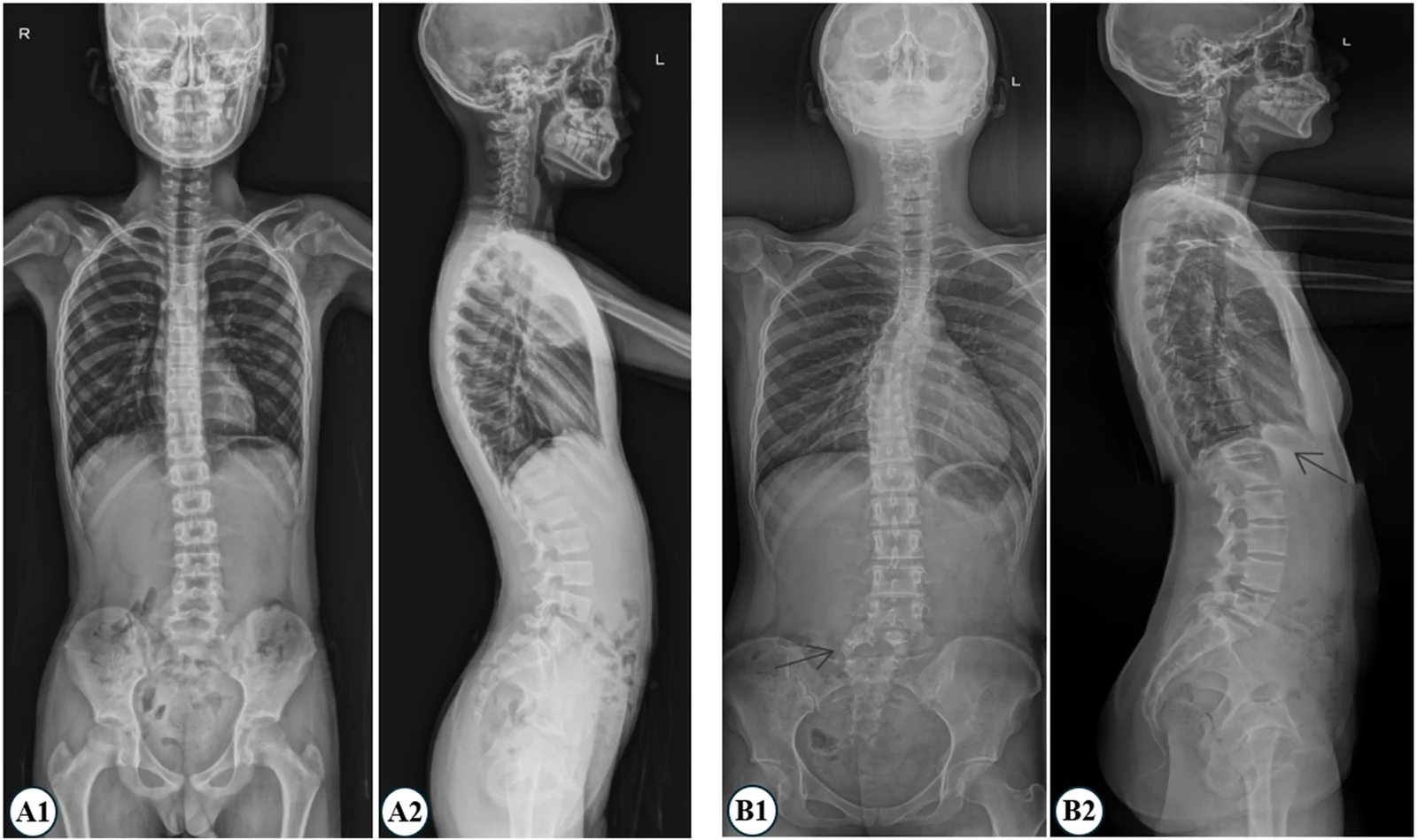
Representative full-spine radiographs obtained with a large-area flat-panel detector (FPD) and a conventional FPD. **(A1, A2)**, anteroposterior and lateral radiographs acquired with the large-area FPD in a single exposure (image quality score, 4 for both). **(B1, B2)**, anteroposterior and lateral radiographs acquired with the conventional FPD by segmented acquisition and image stitching (image quality score, 1 for both). Arrows indicate stitching misregistration artifacts.

The DRX group had a significantly shorter examination duration than the conventional group (3.63 ± 0.83 min vs. 11.59 ± 2.31 min, *P* < 0.001). Subjective image quality scores were analyzed as ordinal data. For the patient-level analysis, the median score across the four reviewers was used. Scores were significantly higher in the DRX-LC group than in the conventional detector group [median [IQR], 4 [3–4] vs. 3 [2.5–3]; *P* < 0.001] (Table 1). Inter-rater agreement among the four reviewers was excellent (Kendall's W = 0.918; *P* < 0.001). Reviewers consistently noted clearer visualization of vertebral cortices and trabecular structures and fewer artifacts in the DRX group, in which no stitching artifacts were observed. Representative images are shown in Figure 2.

### Exposure parameters

Compared with the conventional group, the DRX group showed significantly lower exposure requirements in both projections (Table 1). Specifically, the AP mAs was 39.12 ± 1.16 in the DRX group versus 70.43 ± 10.45 in the conventional group (*P* < 0.001), and the LAT mAs was 54.56 ± 3.02 versus 76.69 ± 12.83, respectively (*P* < 0.001). Accordingly, cumulative mAs was also significantly lower in the DRX group than in the conventional group (93.88 ± 3.59 vs. 146.89 ± 21.82, *P* < 0.001).

## Discussion

This retrospective clinical comparison showed that the DRX-LC large-area FPD–based imaging system was associated with a significantly shorter examination duration than the conventional FPD system (3.63 ± 0.83 min vs. 11.59 ± 2.31 min, *P* < 0.001). The DRX-LC system also required lower cumulative tube current-exposure time product values (93.88 ± 3.59 mAs vs. 146.89 ± 21.82 mAs, *P* < 0.001) and achieved higher subjective image quality scores [median [interquartile range], 4 (3, 4) vs. 3 [2.5–3], *P* < 0.001]. Taken together, these system-level findings suggest that, under the evaluated standardized protocol, single-exposure large-area imaging may improve workflow efficiency and clinical image acceptability.

The present findings should be interpreted in the context of prior international studies of long-length imaging. Dietrich et al. reported shorter whole-spine examination times with a biplanar slot-scanning system than with standard digital radiography (248 vs. 449 s) (8), supporting the general workflow advantage of technologies that avoid conventional multistation acquisition. Jeon et al. found that reducing mAs by 50% in full-spine radiography did not significantly affect subjective image quality or Cobb angle measurement (9), emphasizing that clinical image acceptability depends on the overall acquisition and processing protocol rather than exposure parameters alone. Furthermore, Supakul et al. identified potentially clinically relevant stitching errors in 16% of digitally stitched scoliosis examinations (7). These studies support the clinical rationale for minimizing multistation stitched acquisitions; however, they evaluated different imaging systems and protocols and should not be regarded as direct comparators of the present DRX-LC workflow.

Using the workflow-based definition of examination duration, the conventional segmented acquisition and stitching protocol required a mean of 11.59 ± 2.31 min and served as the local reference standard. In comparison, the DRX-LC system reduced the mean examination duration to 3.63 ± 0.83 min, representing an absolute reduction of 7.96 min (68.7%). This reduction is clinically meaningful because conventional full-spine radiography requires repeated segmented exposures followed by image stitching, which prolongs the examination and increases susceptibility to patient motion and intersegment misregistration. By eliminating the need for 4–6 repeated acquisitions, the DRX-LC system simplified the acquisition workflow and reduced opportunities for motion-related degradation (14–18) This advantage may be especially relevant in patients who have difficulty maintaining a stable posture during prolonged standing examinations.

Importantly, the lower mAs values observed in the DRX group did not come at the expense of image quality. Instead, subjective image quality improved, with clearer cortical and trabecular depiction and fewer artifacts. However, objective image quality metrics, such as signal-to-noise ratio and contrast-to-noise ratio, were not available; therefore, the subjective findings could not be directly compared with an objective image quality evaluation tool. This finding is consistent with the broader concept that exposure level alone does not determine perceived or diagnostic image quality. Tan et al. reported that half-dose total-body positron emission tomography/computed tomography yielded image quality scores that were even higher than those of a conventional full-dose protocol (19), indicating that image quality depends on multiple factors, including detector performance, signal processing, and post-processing strategy. The present study extends this concept to digital radiography. Although large-area FPDs may theoretically be affected by changes in signal-to-noise ratio and detective quantum efficiency, we did not observe an apparent image-quality penalty in the DRX-LC group (10, 20). Smart Noise Cancellation (SNC), an optional deep convolutional neural network–based image-processing module in Carestream's Eclipse/ImageView platform, was enabled in the DRX-LC imaging workflow (21). Therefore, SNC may have contributed to the lower apparent noise and higher subjective image quality scores observed in the DRX-LC group; however, its independent effect was not measured in the present study. Nevertheless, the specific contribution of this algorithm to full-spine digital radiography was not directly assessed in the present study and warrants further investigation.

At the same time, clinical implementation of large-area FPDs requires attention to the trade-off between expanded anatomical coverage and scatter control. In full-spine radiography, the larger irradiated field may increase the amount of scattered radiation reaching the detector, potentially reducing image contrast and signal-to-noise ratio if not adequately controlled by collimation, antiscatter grids, or image processing (10, 20). Because scatter, signal-to-noise ratio, and detective quantum efficiency were not quantitatively measured in this study, these technical factors could not be separately evaluated. In the present study, operator-related variation was minimized because all examinations were performed by trained radiologic technologists using a standardized protocol, with automatic exposure control applied in both groups and fixed tube voltages for AP and LAT imaging. In routine clinical practice, however, technologists may modify exposure settings according to patient body habitus or clinical requirements; such adjustments may affect tube current-exposure time product values and image quality (20, 22). This issue may be particularly relevant when lower tube voltage is used or in patients with larger body habitus. Lower tube voltage may reduce x-ray penetration, whereas greater patient thickness can attenuate the primary beam and increase scattered radiation; both factors may impair detector signal and image contrast. In such settings, maintenance of diagnostic image quality may require optimization of acquisition and processing parameters, including appropriate adjustment of tube voltage or automatic exposure control settings, tight collimation, antiscatter-grid use, and, where available, carefully validated noise-reduction processing (15, 23). In our study, protocol standardization reduced human-factor variability; however, future studies should further explore automated exposure control and intelligent dose-optimization approaches for large-area FPD imaging in order to achieve a more dynamic balance between exposure parameter settings and image quality (17, 22, 24).

The clinical importance of these findings lies in providing a practical optimization strategy for full-spine digital radiography. The single-exposure capability of a large-area FPD may streamline workflow, reduce repeated positioning, and lessen motion or stitching artifacts associated with multistation imaging (2, 3). More broadly, innovation in spinal imaging is moving toward simultaneous dose reduction and preservation of diagnostic performance. Related examples include deep learning-assisted radiation-free virtual x-ray assessment of scoliosis (25) and comparative studies showing that the dose-saving potential of emerging detector technologies should be verified through rigorous image-quality assessment rather than assumed *a priori* [22]. In the future, integration of artificial intelligence-based image-quality assessment may further improve evaluation efficiency, reduce subjective bias, and facilitate more individualized optimization of full-spine radiographic protocols (17, 24).

This study also has several limitations. First, it was conducted at a single center with a modest sample size. Second, image quality assessment was based on subjective scoring, although evaluation by multiple experienced reviewers was used to improve robustness. Third, standardized patient dosimetry metrics, such as dose-area product, and objective image quality metrics, such as signal-to-noise ratio and contrast-to-noise ratio, were not available. Fourth, beam uniformity and geometric distortion associated with the acquisition geometry, particularly the 180-cm source-to-image distance used for lateral imaging, were not quantitatively evaluated. Therefore, we cannot exclude geometry-related spatial nonuniformity or distortion, and our findings should be limited to the observed workflow and subjective clinical image-quality outcomes rather than interpreted as evidence of preserved geometric accuracy. Prospective phantom and patient studies incorporating quantitative geometric and uniformity measurements are needed. Fifth, the DRX-LC system incorporated vendor-specific image processing, including Smart Noise Cancellation, whereas the conventional imaging pipeline did not use matched post-processing. Therefore, the effects of detector size, acquisition geometry, scatter conditions, and image-processing software could not be separated. The observed image quality differences should be interpreted as system-level findings rather than evidence of an independent effect of detector size. Finally, subgroup analyses according to body habitus, spinal pathology, or age were not performed. Future multicenter studies incorporating quantitative dosimetry and objective image quality analysis are warranted.

Despite these limitations, the present findings support the clinical value of large-area FPDs for full-spine digital radiography. The ability to complete full-spine imaging in a single exposure may streamline workflow, reduce repeated positioning, and improve diagnostic image consistency. Further studies may also explore integration with automated image-quality assessment and dose-optimization algorithms to maximize the benefits of this acquisition strategy.

## Conclusion

Compared with conventional flat-panel detector imaging requiring segmented acquisition, the DRX-LC large-area detector–based imaging system was associated with fewer acquisitions, shorter examination time, lower tube current-exposure time product values, and higher subjective image quality These results support its use as a practical option for optimizing full-spine radiographic protocols.

## Data Availability

The raw data supporting the conclusions of this article will be made available by the authors, without undue reservation.
